# Glycemic Effects and Safety of L-Glutamine Supplementation with or without Sitagliptin in Type 2 Diabetes Patients—A Randomized Study

**DOI:** 10.1371/journal.pone.0113366

**Published:** 2014-11-20

**Authors:** Dorit Samocha-Bonet, Donald J. Chisholm, Fiona M. Gribble, Adelle C. F. Coster, Kevin H. Carpenter, Graham R. D. Jones, Jens J. Holst, Jerry R. Greenfield

**Affiliations:** 1 Diabetes and Metabolism Division, Garvan Institute of Medical Research, Sydney, Australia; 2 Faculty of Medicine, UNSW (The University of New South Wales), Sydney, Australia; 3 Cambridge Institute of Medical Research and Department of Clinical Biochemistry, University of Cambridge, Cambridge, United Kingdom; 4 School of Mathematics and Statistics, UNSW (The University of New South Wales), Sydney, Australia; 5 Disciplines of Genetic Medicine & Paediatrics and Child Health, University of Sydney, Sydney, Australia; 6 Department of Chemical Pathology, St. Vincent's Hospital, Sydney, Australia; 7 NNF Center for Basic Metabolic Research, Department of Biomedical Sciences, University of Copenhagen, Panum Institute, Copenhagen, Denmark; 8 Department of Endocrinology and Diabetes Center, St. Vincent's Hospital, Sydney, Australia; University of Tolima, Colombia

## Abstract

**Background and Aims:**

L-glutamine is an efficacious glucagon-like peptide (GLP)-1 secretagogue *in vitro*. When administered with a meal, glutamine increases GLP-1 and insulin excursions and reduces postprandial glycaemia in type 2 diabetes patients. The aim of the study was to assess the efficacy and safety of daily glutamine supplementation with or without the dipeptidyl peptidase (DPP)-4 inhibitor sitagliptin in well-controlled type 2 diabetes patients.

**Methods:**

Type 2 diabetes patients treated with metformin (n = 13, 9 men) with baseline glycated hemoglobin (HbA1c) 7.1±0.3% (54±4 mmol/mol) received glutamine (15 g bd)+ sitagliptin (100 mg/d) or glutamine (15 g bd) + placebo for 4 weeks in a randomized crossover study.

**Results:**

HbA1c (*P* = 0.007) and fructosamine (*P* = 0.02) decreased modestly, without significant time-treatment interactions (both *P* = 0.4). Blood urea increased (*P*<0.001) without a significant time-treatment interaction (*P* = 0.8), but creatinine and estimated glomerular filtration rate (eGFR) were unchanged (*P*≥0.5). Red blood cells, hemoglobin, hematocrit, and albumin modestly decreased (*P*≤0.02), without significant time-treatment interactions (*P*≥0.4). Body weight and plasma electrolytes remained unchanged (*P*≥0.2).

**Conclusions:**

Daily oral supplementation of glutamine with or without sitagliptin for 4 weeks decreased glycaemia in well-controlled type 2 diabetes patients, but was also associated with mild plasma volume expansion.

**Trial Registration:**

ClincalTrials.gov NCT00673894

## Introduction

Impaired insulin secretion contributes to hyperglycemia in type 2 diabetes. Glucagon-like peptide (GLP)-1 is secreted from intestinal L-cells in response to nutrients and is rapidly degraded by dipeptidyl peptidase (DPP)-4. GLP-1 contributes to myriad of metabolic effects, including insulin secretion, beta cell proliferation, slowing of gastric emptying and increased satiety; all are desirable features of type 2 diabetes therapy [1]–[3]. GLP-1 receptor agonists and DPP-4 inhibitors are used to treat type 2 diabetes patients. However, enhancement of GLP-1 secretion with meals has the advantage of increasing GLP-1 concentrations in the intestinal milieu, where it is believed to act on vagal neurons and mediate its central effects. Moreover, the cleaved GLP-1 product (9–36), which suppresses hepatic glucose production and exerts antioxidant action [4], may also be enhanced by agents that increase GLP-1 secretion.

The amino acid L-glutamine is an efficacious GLP-1 secretagogue for the colonic L-cells model GLUTag [5] and primary murine colonic culture [6]. Well-controlled type 2 diabetes patients have intact GLP-1 response to glutamine ingestion [7] and glutamine (30 g) or glutamine (15 g) in combination with the DPP-4 inhibitor sitagliptin (100 mg) decreases postprandial glycaemia and increases circulating insulin and GLP-1 when administered with a meal [8].

The primary aim of this randomized crossover study was to determine the glycemic effect of 4 weeks of glutamine (15 bd) supplementation with sitagliptin (100 mg/d) or placebo in type 2 diabetes patients treated with metformin. We hypothesized that both treatments will decrease hemoglobin A1c (HbA1c) and fructosamine, with a greater effect exhibited in the combined glutamine-sitagliptin treatment, due to more pronounced postprandial increases in GLP-1 in the circulation. The secondary aim was to evaluate the safety of glutamine supplementation.

## Methods

This study was conducted according to the principles expressed in the declaration of Helsinki. The study was approved by the Human Research and Ethics Committee at St Vincent's Hospital, Sydney. All participants gave written informed consent prior to commencement of the study. The protocol for this study and supporting CONSORT checklist are available as supporting information (Checklist S1 and Protocol S1). The study was registered at www.ClinicalTrials.gov (NCT00673894).

### Participants

**Figure 1:**
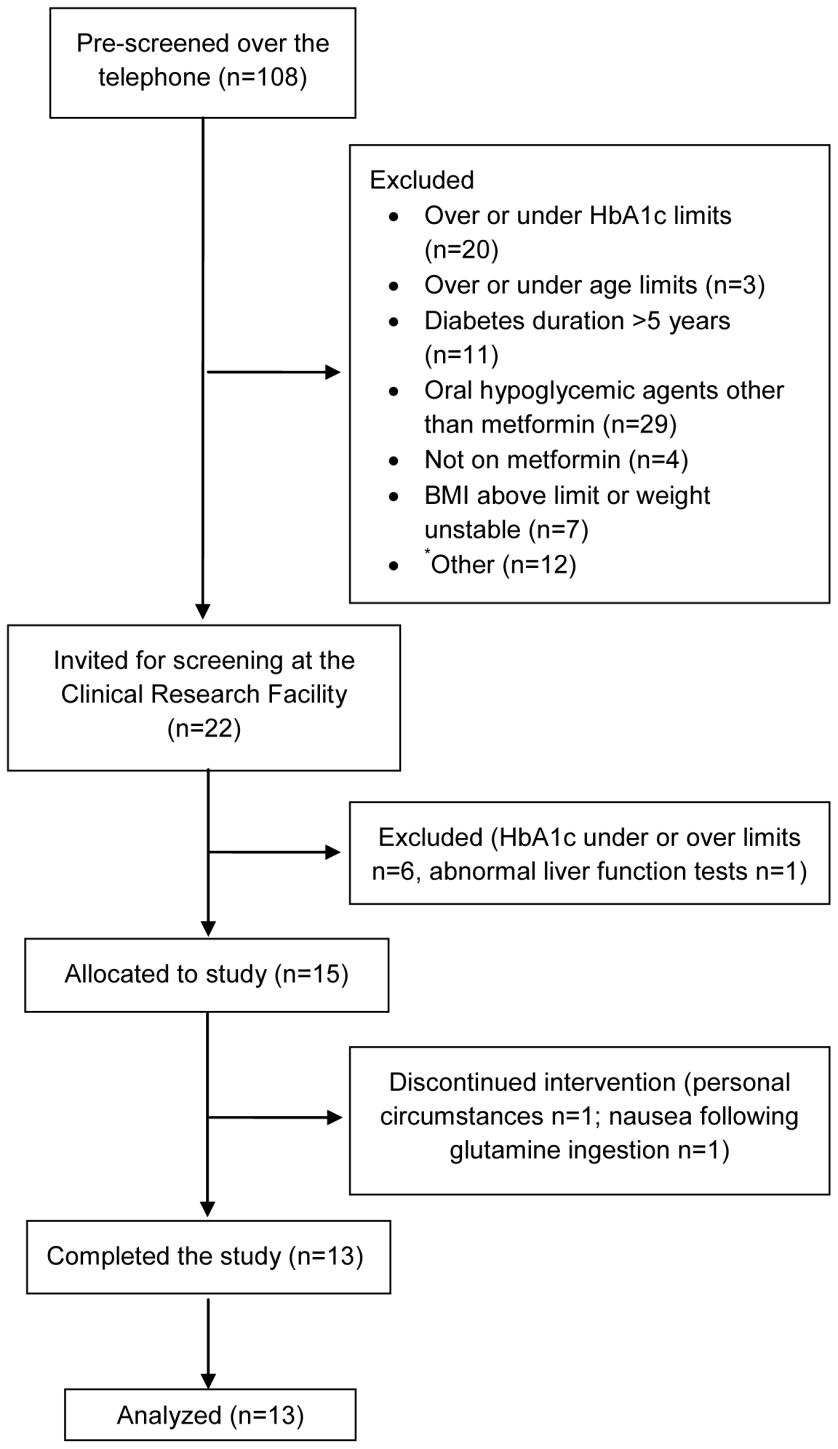
Flow of participants in the study. ^*^Other reasons include participant not able to take time off work or other engagements (n = 3), family not approving (n = 2), underlying disease (n = 3), weight unstable or actively trying to lose weight (n = 2), change of mind (n = 1) and inability to provide informed consent (n = 1).

Type 2 diabetes patients were recruited through advertisements at the St Vincent's Hospital precinct, Sydney and in local newspapers. Participants were recruited and followed between January 2010 and November 2011. Inclusion criteria were age 40–70 years, short diabetes duration (5 years or less), treatment with metformin in a stable dose (≤2000 mg/d) for at least 3 months, HbA1c 6.5–9% (48–75 mmol/mol), BMI ≤40 kg/m^2^ and stable body weight in the preceding 6 months (±2 kg). Exclusion criteria were treatment with oral hypoglycemic agents other than metformin, ethanol intake of 40 g/d or more, liver or kidney disease or abnormal full blood count, renal or liver function tests, use of weight loss medications, previous bowel surgery or documented malabsorption. Of the 108 individuals pre-screened for eligibility over the telephone, 22 were invited to a screening visit at the clinic. Fifteen participants (Caucasian, 10 men) were included and 13 (9 men) completed the study (Figure 1).

### Trial design

**Figure 2:**
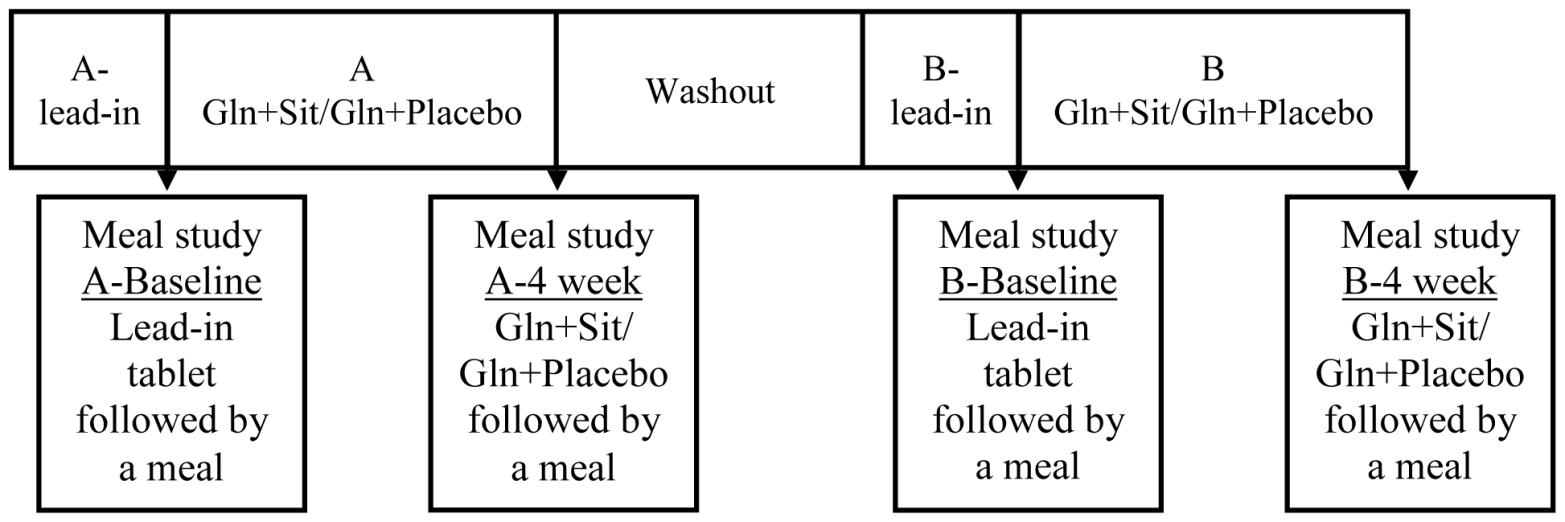
Study timeline. The effect of glutamine with sitagliptin or placebo was assessed in a randomized crossover study. Each study period (A and B) was initiated with 2-weeks lead-in followed by L-glutamine (Gln) 15 g with breakfast and dinner with either sitagliptin (Sit; 100 mg) or placebo for 4 weeks. Participants visited the Clinical Research Facility at day 0 (Baseline) and 4-weeks of studies A and B for a meal challenge. Treatments were randomly assigned with a 4–6 week washout period between A and B studies.

The primary endpoints of this study were the glycemic control markers HbA1c and fructosamine, determined as below (Laboratory Analyses). Two treatments were considered in the study and randomized crossover design was applied (Figure 2). Participants received glutamine (15 g bd) with sitagliptin (100 mg) or placebo. Treatment allocation order was randomized according to a randomization sequence provided by Merck. A nurse not involved directly with the study was in charge of distribution of sequentially numbered containers of sitagliptin or placebo to participants and kept a list with participants' identification numbers and treatment sequence allocation. Study participants, investigators and nurses were blinded to the treatment allocation order. HbA1c and fructosamine levels were determined at baseline (control). Participants were then administered their first treatment over a 4 week period. Endpoint levels were then determined. This period was followed by a 4–6 week washout period. The second treatment trial then followed using the same protocol.

### Procedures

Screening study was performed at the Clinical Research Facility at the Garvan Institute of Medical Research and included weight, height and blood pressure measurements, and medical examination. Blood was drawn for renal and liver function tests, full blood count, and HbA1c. Participants were provided with lead-in placebo tablets and instructed to take 1 tablet 15 min before breakfast for two weeks preceding each baseline study (lead-in, Figure 2). On day 0 and 4-weeks of each study participants attended the Clinical Research Facility fasting from 22:00 h the previous night for a meal challenge (Figure 2). Weight, height and blood pressure were measured and indirect calorimetry performed following 30 min of supine rest (Parvomedics, UT, USA). Heart rate was measured by a SphygmoCor device (AtCor Medical, Australia). A large-bore intravenous indwelling cannula was inserted into a large antecubital vein for blood sampling. Blood was collected for glycemic markers, full blood count, liver and renal function tests and glutamine. After drawing blood twice 10 min apart at baseline for blood glucose, serum insulin and plasma GLP-1 measurements, the lead-in placebo tablet on day 0 or the tablet taken during the study on the 4-weeks visits was taken in 300 ml of cold water and followed by a low fat meal comprising 33 g Wheat-Bix (Sanitarium) and 250 mL low fat milk, providing 230 kcal (37 g carbohydrate, 1.3 g fat and 16 g protein), as described previously [8]. Meal was consumed within 8 min and blood samples were drawn at 15, 30, 45, 60, 90, 120, 150 and 180 min. At completion of A-Baseline and B-Baseline studies (Figure 2), participants were provided with 4 weeks' worth of study tablets and L-glutamine (Cambridge Commodities, Cambridge, United Kingdom) and instructed to fill compliance lists and diet diaries. At home, during A and B studies, participants took sitagliptin (100 mg) or placebo 15 min prior to L-glutamine (15 g), followed by their normal breakfast. L-glutamine (15 g) was taken again 15 min prior to dinner. Meal challenge at 4 weeks of A and B studies was repeated, but participants received not only the tablet they had been taking in the preceding 4 weeks, but also glutamine (15 g) followed by the meal (Figure 2).

### Compliance, diet and physical activity monitoring

Participants were instructed not to change their eating habits and physical activity levels during the study to avoid confounding effects of diet and physical activity on glycemic control. They recorded food and drinks consumed during 6 days in the lead-in and study periods, filled daily compliance lists and were contacted by the research nurse weekly. On clinic study days, participants returned residual tablets and glutamine, and intake of protein-rich food items and physical activity level were assessed by 7-day questionnaires.

### Laboratory analyses

HbA1c was analyzed by cation-exchange HPLC using the Variant II analyzer (Bio-Rad Laboratories, Gladesville, NSW, Australia), fructosamine by a nitro-blue tetrazolium method, fasting plasma glucose by a hexokinase method and other biochemistry tests using standard methods on a Roche Modular analyzer using Roche reagents (Roche Diagnostics Australia, Castle Hill, NSW Australia). Full blood count was measured using a Beckman Coulter analyzer (Beckman-Coulter, Gladesville NSW, Australia). Whole blood glucose was assayed immediately after collection by the glucose oxidase electrode (Yellow Springs Instrument Company; Life Sciences). Insulin was measured in sera stored at −80°C by radioimmunoassay (Millipore, St Charles, USA). Blood samples for plasma glutamine were collected into chilled heparin-coated tubes and immediately centrifuged (7 min at 4100 × g), snap frozen and stored at −80°C until analysis. Plasma glutamine was measured by reverse phase ultra-performance liquid chromatography with UV detection (Acquity UPLC, Waters, Rydalmere, NSW, Australia). Plasma was deproteinized with 10% s-sulphosalicylic acid containing norvaline as an internal standard. Amino acids were then derivatized with 6-aminoquinolyl-N-hydroxysuccinimidyl carbamate and separated by gradient elution. Blood samples for total and active GLP-1 were collected into chilled EDTA-coated tubes (with DPP-4 inhibitor and trasylol in the active GLP-1 testing tube) and immediately centrifuged (7 min at 4100 × g), snap-frozen and stored at −80°C until analysis. Total and active GLP-1 concentrations were measured as previously described [8]. Briefly, total GLP-1 was measured by radioimmunoassay after extraction of plasma with 70% ethanol (v:v, final concentration). Carboxy-terminal GLP-1 immunoreactivity was determined using antiserum 89390, which has an absolute requirement for the intact amidated carboxyl terminus of GLP-1 7–36 amide and cross-reacts <0.01% with carboxy-terminally truncated fragments and 89% with GLP-1 9–36 amide, the primary metabolite of DPP-4-mediated degradation. The sum of the two components, total GLP-1 concentration, reflects the rate of secretion of the L-cell [9]. Active GLP-1 was analyzed using an enzyme-linked immunosorbent assay on unextracted plasma, as reported previously [8].

### Statistical analysis

Area under the curve (AUC) of postprandial glucose, insulin, insulin-to-glucose ratio, total and active GLP-1 concentrations were calculated using the trapezoidal rule. Insulin data were log10-transformed prior to statistical analysis. All values are presented as means ± standard deviation (SD), unless stated otherwise. An a priori power analysis for the ANOVA repeated measures within-between interaction design (α = 0.05, power  = 0.8) was performed (G*Power version 3.0.10) giving a minimum sample size of 6. The effects of the 4 week supplementation of glutamine with sitagliptin or glutamine with placebo were analyzed using repeated measure ANOVA. For this, the within subject factor was time – detecting differences in measured endpoints at 4 weeks supplementation compared to baseline. The between subjects factor was the treatment (supplementation) type – either glutamine with sitagliptin or glutamine with placebo. The significance (*P*-values) for both the within subject factor (time), and the interaction of time and treatment is reported. Normal probability plots of the *P*-values versus their rank were then examined to determine statistical significance (not shown). Relationships that were found to be non-linear were indicative of statistically significant results. The data below *P* of 0.05 were in the non-linear regions. Data were analyzed using SPSS version 21.

## Results

### Cohort characteristics

Participants were 65±6 years old with BMI 28±4 kg/m^2^ on average. Type 2 diabetes duration was 3.3±1.7 years, HbA1c 7.1±0.3% (54±4 mmol/mol), fasting plasma glucose 7.1±1.0 mmol/L and glutamine 572±67 µmol/L (range 444–677 µmol/L).

### The effects of the treatments on HbA1c, fructosamine, fasting plasma glucose and GLP-1

HbA1c and fructosamine decreased modestly, without significant time-treatment interactions. Fasting plasma glucose did not change significantly with the treatments, but there was a significant time-treatment effect (Table 1).

**Table 1:** Effects of the treatments on glycemic markers, weight and blood pressure.

	Glutamine + Placebo		Glutamine + Sitagliptin		*P* value^1^	
	Baseline	4-weeks	Baseline	4-weeks	Time	Time-Treatment interaction
HbA1c (%)	7.0±0.4	6.9±0.4	6.9±0.3	6.7±0.3	**0.007**	0.4
HbA1c (mmol/mol)	53±4.3	52±4.3	52±3.6	50±3.6		
Fructosamine (µmol/L)	258±40	244±41	246±32	240±30	**0.02**	0.4
Fasting plasma glucose (mmol/L)	7.1±0.8	7.3±0.9	7.2±1.0	6.7±0.8	0.1	**0.003**
Weight (kg)	82.8±15.7	82.9±15.6	82.9±15.1	82.7±15.3	0.8	0.4
RMR (kcal/d)	1510±300	1480±320	1490±330	1540±400	0.8	0.3
Respiratory quotient	0.80±0.04	0.81±0.03	0.81±0.04	0.81±0.03	0.7	0.5
Systolic blood pressure (mmHg)	138±17	137±11	139±16	134±9	0.2	0.5
Diastolic blood pressure (mmHg)	84±9	84±7	84±12	79±6	0.2	0.2
Heart rate (bpm)	71±8	67±9	71±10	69±6	**0.02**	0.4

Data are means ± SD.

RMR, resting metabolic rate.

1 Significance of the repeated measure ANOVA with time (within subjects factor) and the interaction of time with treatments (the between subjects factor) is given (n = 13). Bold values represent *P*<0.05. Normal probability plots of the *P*-values versus their rank were examined to determine statistical significance. Relationships that were found to be non-linear were indicative of statistically significant results. The data below *P* of 0.05 were in the non-linear regions.

Fasting total GLP-1 increased (4.5±2.8 and 6.5±2.3 pmol/L with glutamine + placebo and 5.3±2.6 and 7.4±2.3 pmol/L with glutamine + sitagliptin at baseline and 4-weeks respectively, *P* = 0.006), without a significant time-treatment interaction (*P* = 1). Fasting active GLP-1 also increased with both treatments (0.4±0.6 and 1.0±1.2 pmol/L with glutamine + placebo and 0.8±1.0 and 2.3±1.6 pmol/L with glutamine + sitagliptin at baseline and 4 weeks, respectively, *P*≤0.001), without a significant time-treatment interaction (*P* = 0.1).

### The effect of the treatments on postprandial blood glucose, serum insulin and plasma total and active GLP-1

**Figure 3:**
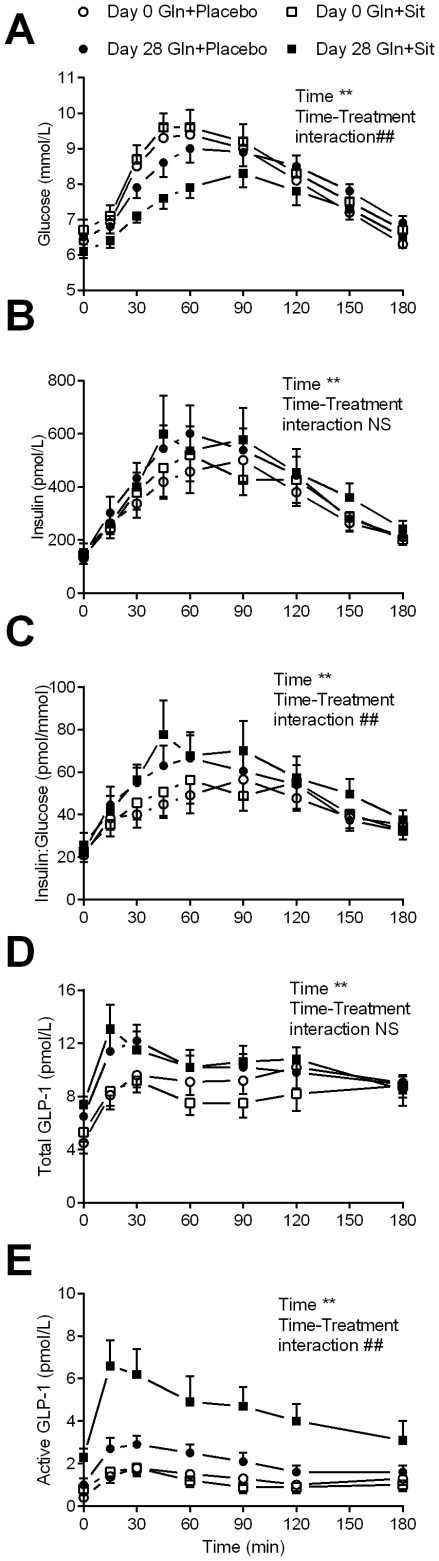
The effect of glutamine + sitagliptin and glutamine + placebo on postprandial circulating concentrations of glucose, insulin and glucagon-like peptide-1. Postprandial circulating concentrations of blood glucose (A), serum insulin (B), insulin to glucose ratio (C) and plasma total and active GLP-1 (D and E, respectively) at baseline (day 0; empty symbols) and 4-weeks (dark symbols) of glutamine + sitagliptin (Gln + Sit; squares) and glutamine + placebo (Gln + Placebo; circles) in type 2 diabetes patients (n = 13). Data are means ± SEM. Significance of the repeated measure ANOVA with time (within subjects factor, ^**^ *P*<0.01) and the interaction of time with treatments (the between subjects factor, ^##^ *P*≤0.01) in the AUC is given (n = 13).

Postprandial glucose AUC decreased (*P* = 0.008), with a significant time-treatment effect (*P* = 0.003, Figure 3A). Postprandial insulin AUC increased (*P*<0.001), without a significant time-treatment effect (*P* = 1, Figure 3B). Insulin-to-glucose AUC increased (*P* = 0.001), with a significant time-treatment effect (*P* = 0.003, Figure 3C). Postprandial total GLP-1 AUC increased (*P* = 0.008, Figure 3D), without a significant time-treatment effect (*P* = 0.2) and active GLP-1 AUC increased (*P*<0.001), with a significant time-treatment effect (*P* = 0.001, Figure 3E).

### The effects of the treatments on weight, blood pressure, full blood count, electrolytes and renal and liver function

Weight, resting metabolic rate (RMR), respiratory quotient (RQ), systolic and diastolic blood pressure were unchanged. Resting heart rate decreased, without a significant time-treatment effect (Table 1). WBC, RBC, Hb and Hct all decreased, without significant time-treatment interactions (Table 2). There were no effects on the red blood cell characteristics mean corpuscular volume (MCV), mean corpuscular Hb (MCH), mean corpuscular Hb concentration (MCHC) and red cell distribution width (RDW; Table 2). Neutrophil count decreased, without significant time-treatment interaction and lymphocyte count decreased with a significant time-treatment interaction (Table 2). Total protein, albumin, alkaline phosphatase and gamma-glutamyl transpeptidase (GGT) all decreased, without significant time-treatment interactions, while alanine transaminase (ALT), aspartate transaminase (AST) and bilirubin were unchanged (Table 2). There were no significant changes in electrolytes, including sodium, potassium, chloride and bicarbonate (Table 2). Blood urea increased, without a significant time-treatment interaction, but creatinine, and therefore estimated glomerular filtration rate (eGFR), were unchanged (Table 2).

**Table 2:** Effects of the treatments on full blood count, renal and liver function and plasma electrolytes.

Marker (normal range)	Glutamine + Placebo		Glutamine + Sitagliptin		*P* value^1^	
	Baseline	4-weeks	Baseline	4-weeks	Time	Time-Treatment interaction
WBC (4.0–11.0 .10^9^/L)	6.2±1.5	5.8±1.2	6.3±1.5	5.5±1.8	**0.004**	0.4
RBC (4.5–6.5 .10^12^/L)	4.3±0.4	4.1±0.3	4.3±0.3	4.1±0.3	**0.003**	0.7
Hemoglobin (130–180 g/L)	135±13	129±10	134±12	129±11	**0.001**	0.8
Hematocrit (0.40–0.54%)	0.40±0.04	0.38±0.03	0.39±0.03	0.38±0.03	**0.001**	0.9
MCV (76–96 fL)	92±4	92±3	92±4	91±4	0.8	0.4
MCH (27.0–32.0 pg)	31.2±1.5	31.3±1.5	31.3±1.4	31.2±1.4	0.6	0.3
MCHC (320–360 g/L)	340±5	341±6	342±5	342±4	0.5	0.7
RDW (11.5–14.5%)	13.3±0.5	13.3±0.6	13.2±0.6	13.3±0.7	0.6	0.9
Platelets (150–400 10^9^/L)	245±66	240±54	252±56	240±58	0.1	0.4
Neutrophils (2.0–7.5 10^9^/L)	3.6±1.0	3.3±0.8	3.7±1.0	3.3±1.5	**0.05**	0.7
Lymphocytes (1.5–4.0 10^9^/L)	1.8±0.6	1.8±0.6	1.9±0.5	1.6±0.5	**0.003**	**0.01**
Monocytes (0.2–1.0 10^9^/L)	0.6±0.2	0.5±0.1	0.5±0.2	0.5±0.1	0.08	0.1
Total protein (60–82 g/L)	70±4	65±5	69±4	67±4	**<0.001**	0.1
Albumin (36–52 g/L)	45±2	43±3	45±2	44±1	**0.02**	0.4
Alkaline phosphatase (30–100 U/L)	62±21	58±17	64±19	57±18	**<0.001**	0.2
GGT (0–35 U/L)	25±13	22±11	25±17	22±13	**0.01**	0.7
ALT (0–30 U/L)	29±11	27±10	26±12	24±8	0.08	0.9
AST (0–30 U/L)	26±8	25±9	23±6	22±6	0.4	1
Total bilirubin (0–18 µmol/L)	11±7	9±4	10±6	10±6	0.2	**0.02**
Sodium (137–146 mmol/L)	142±2	142±2	141±2	141±2	0.5	0.4
Potassium (3.5–5.0 mmol/L)	4.4±0.3	4.4±0.3	4.7±0.3	4.5±0.2	0.2	0.4
Chloride (95–110 mmol/L)	104±3	105±4	104±2	104±2	0.7	0.2
Bicarbonate (24–31 mmol/L)	26±2	26±2	27±2	26±2	0.2	0.9
Urea (3.0–8.5 mmol/L)	6.1±1.1	7.4±2.0	6.4±0.9	7.8±1.5	**<0.001**	0.8
Creatinine (60–120 µmol/L)	76±17	73±13	75±14	76±14	0.6	0.2
eGFR (mL/min/1.73 m^2^)	80±13	81±11	81±10	81±10	0.5	0.6

Data are means ± SD.

WBC, white blood cells; RBC, red blood cells; MCV, mean corpuscular volume; MCH, mean corpuscular hemoglobin; MCHC, mean corpuscular hemoglobin concentration; RDW, red cell distribution width; GGT, gamma-glutamyl transpeptidase; ALT, alanine transaminase; AST, aspartate transaminase.

1 Significance of the repeated measure ANOVA with time (within subjects factor) and the interaction of time with treatments (the between subjects factor) is given (n = 13). Bold values represent *P*<0.05. Normal probability plots of the *P*-values versus their rank were examined to determine statistical significance. Relationships that were found to be non-linear were indicative of statistically significant results. The data below *P* of 0.05 were in the non-linear regions.

### Diet and physical activity during the study

Dietary intake of protein-rich food was not different between baseline and 4-weeks (*P* = 0.6) or between treatment periods (*P* = 0.7). Participants were sedentary and physical activity levels were not different at baseline and 4-weeks (*P* = 0.3) or between treatment periods (243±14 and 245±15 metabolic equivalent of tasks (METs) hr/week for glutamine + placebo and 249±18 and 242±16 METs hr/week for glutamine + sitagliptin at baseline and 4-weeks respectively, *P* = 0.1).

## Discussion

Daily ingestion of L-glutamine, with or without sitagliptin, for 4 weeks decreased HbA1c and fructosamine in well-controlled type 2 diabetes patients treated with metformin. However, glutamine treatment was also associated with modest decreases in concentrations of circulating blood cells, total protein and albumin, without changes in body weight or plasma electrolytes, suggesting mild plasma volume expansion.

Both glycemic control markers, the longer term HbA1c and the shorter term fructosamine decreased significantly with the treatments in the present study, without a significant difference between treatments. These findings suggest that the reduction in glycaemia was attributed to the glutamine. Notably, HbA1c reduction is expected to be larger if treatment was prolonged as the treatment period of 28 days is under the mean red blood cell age of about 50 days [10]. Fructosamine was added to the panel of glycemic markers because of its shorter half-life. The meal studies revealed that the combined glutamine and sitagliptin treatment was more effective in decreasing postprandial glycaemia and in increasing insulin-to-glucose ratio and active GLP-1. Accordingly, we predict that longer treatments would have likely resulted in more pronounced decreases in glycemic control markers with the combined glutamine-sitagliptin treatment. This is also in line with the decreases in fasting plasma glucose only with the sitagliptin combination, potentially attributed to glucagon inhibition [7], [8], [11]. Importantly, reductions in the proportion of HbA1c are unaffected by plasma volume expansion, however decreases in fructosamine may have been affected by the general decreases in circulating proteins in the present study.

Safety of glutamine supplementation of enteral or parenteral nutrition was widely studied in critically-ill patients where L-glutamine is used to maintain intestinal integrity, improve nitrogen balance, prevent infections, decrease oxidative stress and improve survival [12]. Glutamine supplementation in critically-ill patients resulted in conflicting findings, including decreased [12], increased [13] or no effect [14] on complications and mortality rates. Type 2 diabetes has been associated with decreases in circulating glutamine previously [15] but, in this well-controlled diabetic cohort plasma glutamine concentrations were within the reference range [15], [16]. Glutamine supplementation at levels of 1–30 g/d are safe for several hours post ingestion in physically active healthy populations [16] and type 2 diabetes patients [8]. However, safety data of prolonged glutamine intake in non-critically-ill patients are scarce. Galera and colleagues [17] investigated the safety of 14 days of glutamine or casein supplementation in healthy, predominantly sedentary, middle age and elderly individuals in dosages slightly higher than those administered here (0.5 g/kg/d). Unlike the present study, serum creatinine concentrations increased and eGFR decreased with glutamine or casein [17], maybe due to the higher protein intake. Similarly to the present study however, blood urea concentrations increased [17], reflecting the increased dietary nitrogen intake. Interestingly, blood cells, Hb, Hct, total protein and albumin concentrations were all decreased with glutamine in the present study. The decreases in red blood cells without changes in MCV, MCH and MCHC and the general decreases in circulating macromolecules suggest intravascular fluid volume expansion, an effect previously documented in piglets with parenteral glutamine supplementation [18]. Notably, body weight and plasma electrolytes were unchanged in the present study, consistent with minor changes in plasma volume or changes in fluid distribution. The mechanisms involved in extracellular fluid volume expansion with glutamine are unclear, but have been suggested to involve increased glomerular sodium reabsorption [18], which with prolonged glutamine ingestion may alter electrolyte concentrations and acid-base regulation. Furthermore, modest decreases in Hb and Hct were also noted previously with high dose glutamine or casein supplementation for 14 days in healthy individuals [17], suggesting that these effects are not limited to glutamine or type 2 diabetes patients. In any case, further study is required to determine the mechanisms involved in the effect of glutamine on plasma volume expansion.

This is the first study to evaluate the effect of daily glutamine supplementation for a period of weeks on glycemic control and safety in type 2 diabetes patients. There are several strengths to the study, including the sitagliptin versus placebo crossover design that enabled investigation of possible interaction between glutamine and DPP-4 inhibition. Furthermore, the homogeneity of the cohort in terms of age, disease duration and background diabetes treatment increased the power to detect the effects of the treatment on the endpoints. The main limitation of the study is the lack of sitagliptin-alone arm. Furthermore, the relatively low baseline HbA1c and short study duration may have limited the magnitude of change in outcome measures.

In conclusion, daily glutamine administration for 4 weeks decreased HbA1c, irrespective of sitagliptin treatment in well-controlled type 2 diabetes patients treated with metformin. Glutamine administration also resulted in an apparent plasma volume expansion in this population and requires further study.

## Supporting Information

Checklist S1 **Consort checklist.**
(DOC)

Protocol S1 **Trial protocol.**
(PDF)

## References

[1] Drucker DJ (2006) The biology of incretin hormones. Cell Metabolism 3: 153–165. 16517403 10.1016/j.cmet.2006.01.004

[2] Holst JJ, Vilsboll T, Deacon CF (2009) The incretin system and its role in type 2 diabetes mellitus. Mol Cell Endocrinol 297: 127–136. 18786605 10.1016/j.mce.2008.08.012

[3] Nauck MA, Baller B, Meier JJ (2004) Gastric inhibitory polypeptide and glucagon-like peptide-1 in the pathogenesis of type 2 diabetes. Diabetes 53 Suppl 3: S190–196. 10.2337/diabetes.53.suppl_3.s190 15561910

[4] Thomas E, Habener JF (2010) Insulin-like actions of glucagon-like peptide-1: a dual receptor hypothesis. Trend Endocrinol Metab 21: 59–68. 10.1016/j.tem.2009.11.007 PMC4085161 20018525

[5] Reimann F, Williams L, da Silva Xavier G, Rutter GA, Gribble FM (2004) Glutamine potently stimulates glucagon-like peptide-1 secretion from GLUTag cells. Diabetologia 47: 1592–1601. 15365617 10.1007/s00125-004-1498-0

[6] Tolhurst G, Zheng Y, Parker HE, Habib AM, Reimann F, et al (2011) Glutamine triggers and potentiates glucagon-like peptide-1 secretion by raising cytosolic Ca2+ and cAMP. Endocrinology 152: 405–413. 21209017 10.1210/en.2010-0956 PMC3140224

[7] Greenfield JR, Farooqi IS, Keogh JM, Henning E, Habib AM, et al (2009) Oral glutamine increases circulating glucagon-like peptide 1, glucagon, and insulin concentrations in lean, obese, and type 2 diabetic subjects. Am J Clin Nutr 89: 106–113. 19056578 10.3945/ajcn.2008.26362 PMC4340573

[8] Samocha-Bonet D, Wong O, Synnott EL, Piyaratna N, Douglas A, et al (2011) Glutamine reduces postprandial glycemia and augments the glucagon-like peptide-1 response in type 2 diabetes patients. J Nutr 141: 1233–1238. 21593352 10.3945/jn.111.139824 PMC7212026

[9] Orskov C, Rabenhoj L, Wettergren A, Kofod H, Holst JJ (1994) Tissue and plasma concentrations of amidated and glycine-extended glucagon-like peptide I in humans. Diabetes 43: 535–539. 8138058 10.2337/diab.43.4.535

[10] Cohen RM, Franco RS, Khera PK, Smith EP, Lindsell CJ, et al (2008) Red cell life span heterogeneity in hematologically normal people is sufficient to alter HbA1c. Blood 112: 4284–4291. 18694998 10.1182/blood-2008-04-154112 PMC2581997

[11] Solis-Herrera C, Triplitt C, Garduno-Garcia JdJ, Adams J, DeFronzo RA, et al (2013) Mechanisms of Glucose Lowering of Dipeptidyl Peptidase-4 Inhibitor Sitagliptin When Used Alone or With Metformin in Type 2 Diabetes: A double-tracer study. Diabetes Care 36: 2756–2762. 23579178 10.2337/dc12-2072 PMC3747902

[12] Novak F, Heyland DK, Avenell A, Drover JW, Su X (2002) Glutamine supplementation in serious illness: a systematic review of the evidence. Crit Care Med 30: 2022–2029. 12352035 10.1097/00003246-200209000-00011

[13] Heyland D, Drover J, Dhaliwal R (2006) Does the addition of glutamine to enteral feeds affect patient mortality? Crit Care Med 34: 2031–2032 author reply 2032. 16801883 10.1097/01.CCM.0000226837.05375.08

[14] Andrews PJD, Avenell A, Noble DW, Campbell MK, Croal BL, et al (2011) Randomised trial of glutamine, selenium, or both, to supplement parenteral nutrition for critically ill patients. Br Med J 342. 10.1136/bmj.d1542 21415104

[15] Menge BA, Schrader H, Ritter PR, Ellrichmann M, Uhl W, et al (2010) Selective amino acid deficiency in patients with impaired glucose tolerance and type 2 diabetes. Regul Pept 160: 75–80. 19695292 10.1016/j.regpep.2009.08.001

[16] Gleeson M (2008) Dosing and Efficacy of Glutamine Supplementation in Human Exercise and Sport Training. J Nutr 138: 2045S–2049S. 18806122 10.1093/jn/138.10.2045S

[17] Galera SC, Fechine F, Teixeira MJ, Branco Coelho ZC, de Vasconcelos RC, et al (2010) The safety of oral use of l-glutamine in middle-aged and elderly individuals. Nutrition 26: 375–381. 19765954 10.1016/j.nut.2009.05.013

[18] House JD, Pencharz PB, Ball RO (1994) Glutamine Supplementation to Total Parenteral Nutrition Promotes Extracellular Fluid Expansion in Piglets. J Nutr 124: 396–405. 8120659 10.1093/jn/124.3.396

