# Supplementary material for: Glycemic Effects and Safety of L-Glutamine Supplementation with or without Sitagliptin in Type 2 Diabetes Patients—A Randomized Study
Source: PLoS One. 2014 Nov 20;9(11):e113366. doi: 10.1371/journal.pone.0113366 (PMC4239068; doi:10.1371/journal.pone.0113366)
Supplement: Protocol S1 — Trial protocol. (PDF) [file pone.0113366.s002.pdf]

Study Protocol

Version: 5

Date: 18 July 2008

Study title: Effect of glutamine on GLP-1 and insulin secretion in man

Chief Investigator/Supervisor

**name**	**Jerry Greenfield**
title	Dr
qualifications	MB BS BSc(Med) PhD FRACP
positions held	Staff Specialist (Endocrinology), St Vincent's Hospital; Deputy Director, Diabetes Centre, St. Vincent's Hospital; Postdoctoral Research Fellow, Garvan Institute
full mailing address	384 Victoria Street, Darlinghurst 2010
telephone number	(02) 9295 8217
fax number	(02) 9295 8201
e-mail address	j.greenfield@garvan.org.au

Co-Investigator(s), Associate Investigator(s) or Student

**name**	**Donald Chisholm**
title	Prof
full mailing address	384 Victoria Street, Darlinghurst 2010
telephone number	(02) 9295 8205
fax number	(02) 9295 8201
e-mail address	d.chisholm@garvan.org.au

Subjects

On the basis of pilot studies in subjects with T2D, it is predicted that 8 subjects will be required per study group with 80% power and a significance of P=0.05. We will therefore aim to recruit a minimum of 8 lean subjects (Body Mass Index 20 – 25 kg/m^2) and 16 overweight/obese subjects (Body Mass Index 25 – 35 kg/m^2) with T2D. T2D will be defined as a previous diagnosis of T2D (WHO criteria) and/or treatment for T2D. To specifically study patients likely to have substantive residual insulin secretion, only subjects with diabetes duration <4 years will be recruited. Equal numbers of men and women (aged 20 – 75y) will be recruited. Fertile women will undergo urinary pregnancy testing prior to participation. Exclusion criteria will be: smoking, excessive alcohol intake, pregnancy, liver or kidney disease, anaemia, treatment with weight loss agents, weight instability and documented malabsorption.

Background statement: Decreased insulin secretion is a key pathogenic factor in the development of Type 2 diabetes (T2D). Patients with T2D have impaired secretion of glucagon-like peptide-1 (GLP-1), which plays an important role in determining insulin release following a meal. Increasing GLP-1 secretion is therefore a novel approach to improving glycaemia in T2D. *In vitro* data has shown that glutamine is a potent stimulus of GLP-1 secretion. In preliminary data, we have demonstrated that glutamine increases GLP-1 release in obese humans with and without T2D. The studies described below will extend these preliminary data to address whether glutamine is a potential new therapy for the amelioration of hyperglycaemia in T2D.

Hypothesis: Oral glutamine supplementation in T2D will enhance insulin secretion and improve post-prandial glycaemia via stimulation of GLP-1 release.

Specific aims: To determine:

A. The lowest amount of glutamine required to increase circulating insulin levels to a similar extent to that observed following an established insulin secretagogue (sulphonylurea) and to compare the effect of glutamine to alanine and alanylglutamine;

B. Whether glutamine supplementation of a standard mixed meal increases insulin and C-peptide and decreases glucose concentrations in patients with T2D and whether this effect is enhanced by the addition of a DPP-IV inhibitor (to reduce GLP-1 degradation);

C. Whether daily glutamine supplementation has a cumulative effect on GLP-1 secretion in T2D and whether this effect is sustained in the short-term after cessation of glutamine therapy;

D. By comparing oral vs intravenous glutamine administration: (i) whether glutamine-induced insulin secretion is dependent on glutamine absorption from the small intestine into the circulation; and (ii) whether the effect of glutamine on insulin release is direct or whether it is mediated indirectly by GLP-1.

Overall research plan:

Subjects will attend the Clinical Research Facility, Garvan Institute, between 0745 and 0800, having fasted from 2200 the night before the study. Subjects will be instructed not to walk or cycle to study visits. Studies will be performed in a random order on separate days. A large-bore intravenous indwelling cannula will be inserted into an antecubital vein on each visit for blood sampling. During the clamp studies, a second intravenous line in the other arm will be required for the infusion of glucose. At the first visit, weight, height and waist and hip circumferences will be measured. Fasting blood will be drawn for HbA1c, renal and liver function tests and a full blood count. On each study day, 3 fasting blood samples will be taken for glucose, insulin, C-peptide, glucagon and GLP-1. Due to first-pass hepatic extraction of insulin, C-peptide will be measured to give a more precise index of insulin secretion.

Protocol A: Dose-response study

Subjects: 8 lean healthy individuals and 8 patients with T2D. Patients with T2D not treated with oral hypoglycaemic agents or insulin will be recruited for this study.

Study Design: 7 separate fortnightly visits will be required for this study over 3 months. Baseline blood samples will be collected at t = -10, -5 and -1 min. On separate study days, subjects will randomly receive either 5g, 15g or 30g of glutamine (in 300ml of water), alanine (equimolar to 15g of glutamine, in 300ml of water), alanylglutamine (equimolar to 15g of glutamine, in 300ml of water), gliclazide 80mg (in 300ml of water) or water alone (300ml). Alanylglutamine, a stable and highly soluble alanine-glutamine didpeptide [1], may have the advantage of delivering a greater concentration of free glutamine to the distal small intestine. As glutamine is unlikely to be released until the dipeptide is hydrolysed, resistance to hydrolysis in the upper small intestine is likely to result in increased exposure of distal L-cells to ingested glutamine. Alanine will be used as an additional control, as it is the other amino acid component of the dipeptide and only has a moderate effect on GLP-1 secretion *in vitro*. The test substance will be ingested over 2 minutes (last mouthful at t = 0). Blood samples will be collected at t = 15, 30, 45, 60, 90 and 120 min for blood

glucose and plasma GLP-1, insulin, C-peptide and glucagon. Glutamine will be measured at t = -10, 30, 60 and 120 min during the 3 glutamine and the alanylglutamine visits.

Protocol B: Meal supplementation study

Subjects: Lean subjects and 2 groups of patients with T2D: 8 untreated patients (same subjects included in Study A) and 8 treated with metformin alone (500 – 2000 mg/d; >3 months), as metformin has been shown to inhibit DPP-IV activity [2].

Study Design: Subjects will attend for up to 5 fortnightly visits over a period of 1 - 2 months. At each visit, fasting baseline blood samples will be taken for glucose, insulin, C-peptide, glucagon and GLP-1 at t = -70, -65 and -61 min. On different study days, subjects will be randomly assigned to consume: (i) a DPP-IV inhibitor (sitagliptin, Merck ®) at t = -25 min and 300ml of water (at t = -10 min); (ii) glutamine (15g or 30g) in 300ml of water at t = -10 min; (iii) sitagliptin (at t = -25 min) and glutamine (15g in 300ml of water, at t = -10 min) or (iv) no sitagliptin or glutamine (ie. meal alone). On all study days, the test substance (water or glutamine) will be immediately followed by a 'carbohydrate-only' meal (containing predominantly 30 – 40g of carbohydrate). Blood samples will then be collected at t = 0 (end of meal) and at t = 15, 30, 45, 60, 90, 120, 150 and 180 min for glucose, GLP-1, insulin, C-peptide and glucagon. Glutamine will be measured at t = -70, 30, 60 and 120 min on the 2 visits during which glutamine is administered.

Protocol C: Daily supplementation study

Subjects: Subjects with T2D included in Study B will be invited to participate in an unblinded, randomised, placebo-controlled, cross-over study examining the effect of daily (3 week) supplementation of glutamine alone or glutamine plus sitagliptin on GLP-1, insulin, C-peptide, glucagon and glucose levels versus control (water). A minimum of 8 patients with adequate glycaemic control (HbA1c 7 – 8%) will be recruited and no changes in diabetes treatment will be allowed in this time (unless hypoglycaemia occurs, which is not expected with metformin therapy).

Study Design: Subjects will be required to attend the Clinical Research Facility a total of 18 times over ~6 months. Subjects will receive daily supplementation with: (i) glutamine + water for 3 weeks (first dose with lunch on day 0 and last dose with dinner on day 21); (ii) glutamine + water + sitagliptin for 3 weeks; and (iii) water alone for 3 weeks, in a random order, with each treatment period separated by a 4 – 6 week washout period. During active treatment, subjects will be asked to consume a predetermined amount of glutamine powder (determined from the dose-finding study) with all 3 meals. Participants will receive a weekly call from a Research Nurse to assess adherence. In addition, the weight of the glutamine storage container will be covertly recorded at the beginning and end of the glutamine treatment period to assess compliance with glutamine supplementation. During both the active (glutamine and glutamine plus sitagliptin) and placebo (water) treatment phases, glucose, GLP-1, insulin, C-peptide and glucagon responses to a mixed-meal (breakfast, as per Study B) will be examined (at the time-points at baseline (day 0, meal alone), acutely (day 3, meal + glutamine), on day 21 (meal + glutamine) and on day 23 (meal alone, in order to examine whether an enhancement of GLP-1 secretion is sustained in the short term). Glutamine will be measured t = -10, 30, 60 and 120 min following the meals which are preceded by glutamine. To examine the effect of glutamine on first- and second-phase insulin secretion, a 120 min hyperglycaemic clamp will be performed on day –1 (ie. morning before the day of the first dose of glutamine) and on day 22 (ie. morning after the last evening dose of glutamine). Hyperglycaemia (blood glucose level 10.8 mmol/L) will be initiated by a bolus of 25% dextrose and will be maintained at this level by a variable-rate infusion of 20% dextrose for 120 min. The glucose infusion rate will be determined from 5-min blood glucose levels [3]. Plasma insulin levels will be determined at baseline (three fasting samples 5 min apart), 2nd minutely for the first 10 min and every 10 minutes from t = 30 – 120 min. To determine the effect of glutamine on insulin sensitivity, the hyperglycaemic clamp will also be used to calculate insulin sensitivity as previously described [4]. HbA1c and fructosamine levels will be checked on days -1 and 22 to measure overall glycaemic control. To determine whether glutamine supplementation (in part via GLP-1) influences satiety, appetite and food intake, hunger score assessments and 3-day food frequency questionnaires will be completed by all subjects on days -1 and 22 of both treatment periods and subjects will be carefully weighed.

Protocol D: intravenous *vs* oral glutamine study

Subjects: The 8 non-diabetic lean subjects included in study A will be invited to participate in Study D, which will compare the effect of oral vs intravenous (IV) glutamine on insulin secretion.

Study design: Subjects will randomly be given oral glutamine (30g) or a 2-h continuous IV infusion of glutamine on 2 separate study days separated by a minimum of 2 weeks. The rate of IV glutamine infusion will be the amount of IV glutamine required to simulate peak circulating glutamine concentrations achieved following 30g of glutamine orally (931 – 2866 μmol/L). Based on previous experiments in dogs, in which glutamine was infused in isotonic saline solution to achieve concentrations of 1463 ± 139 μmol/L, it is anticipated that a dose of approximately 5 μmol/min/kg body weight will be required [5]. This will be confirmed in a pilot study using glutamine infusion rates of 3, 4 and 5 μmol/min/kg body weight in 2 obese subjects with T2D. All solutions will be prepared on the day of the infusion by the Hospital Pharmacy under sterile conditions. Blood will then be collected as outlined in Study 1.

References

1. Bushen, O.Y., et al., Clin Infect Dis, 2004. **38**(12): p. 1764-70.
2. Green, B.D., et al., Eur J Pharmacol, 2006. **547**(1-3): p. 192-9.
3. Matthews, D.R. and J.P. Hosker, Diabetes Care, 1989. **12**(2): p. 156-9.
4. Wallace, T.M. and D.R. Matthews, Diabet Med, 2002. **19**(7): p. 527-34.
5. Roth, E., et al., Clin Sci (Lond), 1988. **75**(6): p. 641-8.
